# Smart Cementitious Sensors with Nano-, Micro-, and Hybrid-Modified Reinforcement: Mechanical and Electrical Properties

**DOI:** 10.3390/s23052405

**Published:** 2023-02-21

**Authors:** Athanasia K. Thomoglou, Maria G. Falara, Fani I. Gkountakou, Anaxagoras Elenas, Constantin E. Chalioris

**Affiliations:** Department of Civil Engineering, Democritus University of Thrace, 671 00 Xanthi, Greece

**Keywords:** smart cement-mortar sensors, SWCNTs, carbon micro-fibers (CFs), hybrid multiscale reinforcement, mechanical properties, flexural toughness, piezoresistivity, electrical conductivity, impedance, structural health monitoring

## Abstract

The current paper presents the results of an experimental study of carbon nano-, micro-, and hybrid-modified cementitious mortar to evaluate mechanical performance, energy absorption, electrical conductivity, and piezoresistive sensibility. Three amounts of single-walled carbon nanotubes (SWCNTs), namely 0.05 wt.%, 0.1 wt.%, 0.2 wt.%, and 0.3 wt.% of the cement mass, were used to prepare nano-modified cement-based specimens. In the microscale modification, 0.05 wt.%, 0.5 wt.%, 1.0 wt.% carbon fibers (CFs) were incorporated in the matrix. The hybrid-modified cementitious specimens were enhanced by adding optimized amounts of CFs and SWCNTs. The smartness of modified mortars, indicated by their piezoresistive behavior, was investigated by measuring the changes in electrical resistivity. The effective parameters that enhance the composites’ mechanical and electrical performance are the different concentrations of reinforcement and the synergistic effect between the types of reinforcement used in the hybrid structure. Results reveal that all the strengthening types improved flexural strength, toughness, and electrical conductivity by about an order of magnitude compared to the reference specimens. Specifically, the hybrid-modified mortars presented a marginal reduction of 1.5% in compressive strength and an increase in flexural strength of 21%. The hybrid-modified mortar absorbed the most energy, 1509%, 921%, and 544% more than the reference mortar, nano-modified mortar, and micro-modified mortar, respectively. The change rate of impedance, capacitance, and resistivity in piezoresistive 28-day hybrid mortars improved the tree ratios by 289%, 324%, and 576%, respectively, for nano-modified mortars and by 64%, 93%, and 234%, respectively, for micro-modified mortars.

## 1. Introduction

Cement mortar is a commonly used building material with many applications in the construction industry. Many researchers have recently expressed interest in it after its being forced with advanced materials to smartly improve its quasi-brittle behavior and enhance its multifunctional properties for novel construction applications [1,2,3,4,5,6,7]. This goal is achieved by utilizing nanofillers such as carbon nanotubes (CNTs). The CNTs have been studied intensively to improve cementitious materials’ mechanical properties, enhancing mainly post-cracking toughness, ductility, and electrical conductivity by utilizing them as health monitoring sensors. The mortar composites have been multiscale reinforced by carbon fibers or nanotubes [8,9,10,11,12,13,14].

Carbon nanotubes (CNTs) with low densities and high aspect ratios yield higher functionality, increased durability, and electrical conductivity, changing the cement mortar into a self-monitoring sensor. However, the carbon bond developed from van der Waals forces causing inherent agglomerates remains a critical issue in the dispersion process of CNTs into the cementitious matrix [15,16,17,18,19,20,21,22,23,24,25,26]. In order to overcome this obstacle, many researchers have investigated the dispersion of CNTs to create homogenous solutions by handling them with different methods and using different additives as surfactants [27,28]. The surfactant amount normally considerably exceeds the amount of the CNTs by 1.5–10, compared to the CNT amount by weight [29,30]. It has been revealed that the ultrasonication energy-dispersing method may reduce the surfactant amount to 0.5 of the CNTs amount [31]. Sodium dodecylbenzene sulfonate (SDBS), sodium deoxycholate (DOC), cetyltrimethylammonium bromide (CTAB), Pluronic F-127, Gum Arabic, or Triton X were intensively applied, among several surfactants, to achieve the best dispersion of CNTs. Typically, they were used in combination with ultrasonication energy to enhance cementitious composites’ mechanical and electrical properties [32,33,34,35,36,37,38,39,40,41]. In particular, the application of SWCNTs in different proportions in the cement mortar presented an improvement in the compressive strength by 10–21% and the flexural strength by 7–10%, respectively.

The incorporation of carbon fibers (CFs) in the mortar mass provides superior mechanical properties, resulting in innovative composites with exceptional flexural toughness with resistance to crack propagation, ductility, advanced elastic modulus, electrical conductivity, piezoresistivity, as well as corrosion resistance, in the novel lightweight cement composites [42,43,44,45,46,47,48,49,50]. It was investigated that the incorporation of 0.25 wt.% to 4 wt.% carbon fibers yielded the highest 28-day pre-peak and post-crack performance in flexural strength compared to modified mortars incorporating lower CFs concentrations [51]. Other researchers also examined the 0.1 vol.% CNFs and 0.5 vol.% CFs hybrid-modified mortars, presenting a 74% flexural toughness improvement in divergence with only carbon micro-scale fiber specimens [52].

Furthermore, multilevel hybrid reinforcement has also been investigated by several researchers in terms of its beneficial uses in cementitious matrices with sufficient electrical conductivity in contrast to setting carbon fiber and CNTs in separate layers [53,54,55]. However, minimal information exists about the mechanical, electrical, piezoresistive properties, and electrical conductivity of mortar matrix reinforced with multiscale carbon reinforcement combining SWCNTs and CFs, providing a better self-sensing ability and sensitivity. Other scientists also stated that the hybrid combination of carbon nanotubes and microfibers improved the tensile strength by over 14% compared to the raw CFs strengthened specimen [56]. The collaborative interface between the two levels at the nano-/micro-scale of carbon reinforcement results in a synergetic mechanism that prevents the propagation of the nanoscale cracking while increasing the flexural strength by 56% and conductivity by 3 orders of magnitude, compared to plain mortar. Additionally, the hybrid reinforcement provides a post-crack performance, enhancing the toughness and energy absorption ability of hybrid matrices by 10 times higher than the reference mortar until their total failure. The self-sensing ability of cementitious mortars strengthened with conductive carbon nanotubes/nanofibers, or graphene nanoplatelets was investigated in quite a few literature studies [8,57,58,59,60,61,62]. However, a different method was applied to diagnose failure at the pre-peak phases of cementitious beams through the conductance measurements of embedded and surface-joined Piezoelectric lead zirconate titanate (PZT) transducers using a non-portable electromechanical admittance (EMA) monitoring system [63,64,65,66,67,68,69,70,71,72,73].

In our latest research, we examined the most influential factors affecting the multiscale reinforcement, dispersion, and the number of fibers and nanotubes in cement pastes. As a continuation of the existing research, we are expanding the research with the two applied methods of integrating carbon into the mortar matrix: (a) ultrasonic energy for improving the dispersion of the aqueous/SWCNTs solutions and (b) dry mixing to incorporate carbon microfibers [10,11,18]. The novelty of the present research deals with the essential assessment of different amounts of carbon nanotubes (0.05 wt.%, 0.1 wt.%, 0.2 wt.%, and 0.3 wt.%), microfibers (0.05 wt.%, 0.5 wt.%, 1.0 wt.%), and hybrid reinforcement targeting to determine their influence on the mortar matrix’s electromechanical properties, while proving the sensor ability of the optimal reinforcement. Moreover, there is a lack of experimental investigation concerning the slight CNTs concentration in the mortar matrix, under 0.2 wt.% of cement. The preparation of smart mortars and experimental results are first examined in the following sections. The smartness of nano-, micro-, and hybrid-modified mortars is their ability to sense stress and strain changes, which is measured by the average changes in electrical conductivity during the piezoresistive experimental tests. These tests are applied under cyclic compressive loads in the pre-peak phase or monotonic compressive loading up to the modified mortar damage of 2 cm × 2 cm × 8 cm specimens. Flexural toughness and energy absorption capability were determined using the linear elastic fracture model (LEFM) from the crack mouth opening displacement (CMOD) records, while flexure and compressive strength were determined using three-point bending and compressive tests using 4 cm × 4 cm × 16 cm mortar prisms [74,75,76,77,78]. Nyquist plots are used to evaluate the impedance response of nano- and micro-composites [79,80,81,82]. The final section summarizes the assumptions of a comprehensive investigation of the beneficial sensor added to the mortar, comparing and determining the optimal concentrations of the multiscale sensors of the mortar matrix.

## 2. Materials and Methods

The characteristic properties of the single-walled carbon nanotubes (SWCNTs), provided by OCSiAl, are characterized in Table 1. A type I standard Portland cement of 42.5 R was used to cast the cement mortars. For effective dispersion of the carbon nanotubes in the deionized mixing water, nanotube suspensions were made of nanotubes in an aqueous solution that contains the anionic surfactant sodium dodecyl benzenesulfonate (SDBS), with the following chemical composition: C_12_H_25_C_6_H_4_SO_3_Na consisting of numerous organic compounds and commonly called colorless salt, afforded by Aldrich and TBP (Tri-*n*-butyl phosphate). The aqueous/SWCNT/SDBS solutions are dispersed, followed by an ultrasonication process lasting 1 h.

**Figure 1:**
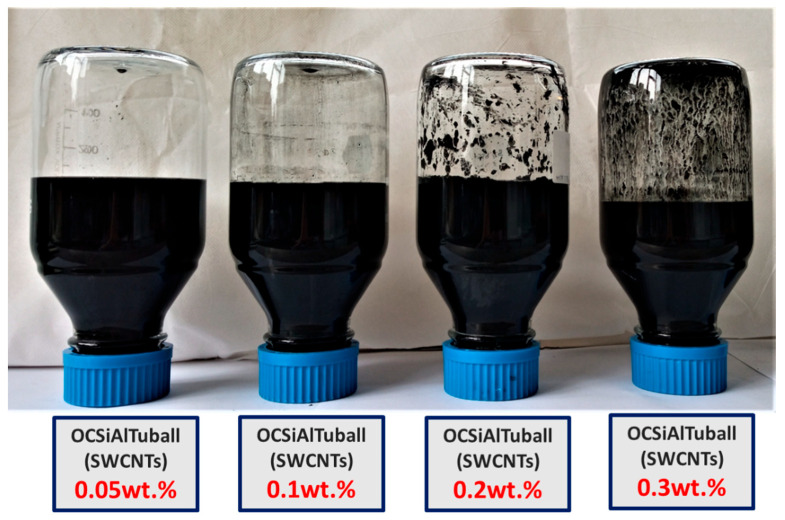
Aqueous suspensions contain chemically modified amounts per weight of cement single-walled carbon nanotubes (SWCNTs).

Figure 1 depicts three types of aqueous suspensions containing chemically modified single-walled carbon nanotubes (OCSial Tuball (SWCNTs) in varying amounts per weight of cement (0.1 wt.%, 0.2 wt.%, and 0.3 wt.%). After an optical observation of the suspensions, the amounts of 0.2 wt.% and 0.3 wt.% SWCNTs show concentrated nanotubes in the form of residue, compared to 0.05 wt.% and 0.1 wt.%.

The 6 mm long microfibers, produced by Toray, were incorporated in cementitious mortar specimens to enhance mechanical and electrical properties. The mixing method was attained according to ASTM C305-20 [83]. The CFs were mixed using the dry mix method with the cement for 2 min, and the TBP was added to the cement and sand mix at a water/cement ratio of w/c = 0.5 and a sand/cement ratio of 3.0. Subsequently, the prepared liquid dispersive system was poured into the mixer and mixed for 2 min. The mixture was stirred quickly for 1 min. This way, CFs are expected to be well dispersed during mixing and then through mold casting.

## 3. Experimental Program

### 3.1. Mechanical Testing Process

**Figure 2:**
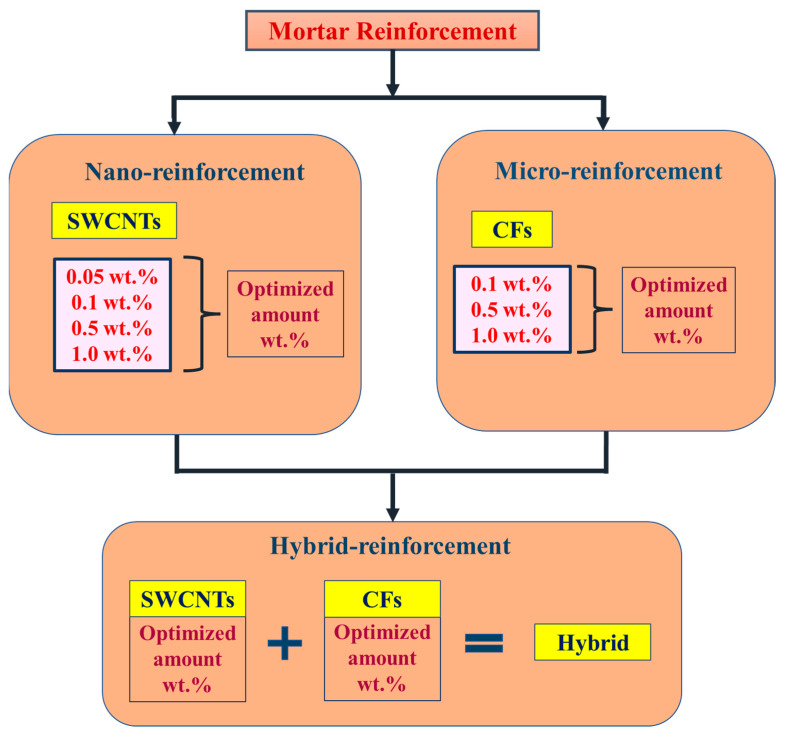
Flowchart of the experimental process of nano-, micro-, and hybrid mortar reinforcement.

The flowchart represents the experimental process of nano-, micro-, and hybrid-modified mortar reinforcement (Figure 2). Specifically, the different amounts of SWCNTs were compared in terms of mechanical properties to achieve an optimum concentration that enhances the strength or energy absorption. The same logic was followed in the micro-reinforcement method. The hybridization was produced by combining the two-scale optimum reinforcements. In the sequel, the electrical properties are meant to be examined in the modified mortars with optimized mechanical properties.

Three-point bending tests were conducted to estimate the mechanical properties (flexural and toughness) of the CFs-strengthened mortars on 4 × 4 × 16 cm prisms at the age of 28 days, following the ASTM C349 standard. The test was performed using a 25 kN MTS hydraulic, closed-loop testing machine with a displacement rate of 0.1 mm/min and the parallel aid of 1 mm strain gauges. The two halves of each prism from the bending test were tested in the compression test. The test was performed with a 2000 kN testing machine under force control with a displacement rate of 0.5 N/min. Notched 2 cm × 2 cm × 8 cm specimens were used to experimentally define the flexural toughness of multi-modified mortars according to the Fracture Mechanics (L.E.F.M.) theory.

### 3.2. Self-Sensing Experimental Process

In addition to the enhanced mechanical strength, carbon multi-reinforced mortars have been widely used for their multifunctional response, derived from their electrical and piezoresistive properties. The electrical conductivity is based on the formation of conductive networks, which are paths created due to the alignment between carbon nanotubes or fibers uniformly dispersed in the mortar matrix or between parallel fibers [8,9,13,18,19,22,61]. This research focuses on combining the two scales (nano and micro) to develop a sensor that, beyond good conductivity and sensing ability, maintains stable mechanical behavior or, even better, improves it. Along with mechanical and electrical responses, the self-sensing ability of multiscale-modified mortar specimens is investigated using conductivity and piezoresistive experimental tests in this paper.

The electrical conductivity, capacitance, and impedance were measured using a HIOKI IM 3533-01 LCR METER (Singapore) at 100 kHz and 2 volts, using the two-pole method with two titanium electrodes embedded in the 28-day mortar matrix in two parallel planes [33]. Considering the dimensions of the hybrid-modified mortars, the conductivity measurements were inverse of the electrical resistivity units, which makes them Ω^−1^ m^−1^ or typically quoted as siemens/meter (S/m). The abovementioned process examined the strain-sensing capability and piezoresistive performance of 28-day modified mortars. The cyclic and monotonic uniaxial compressive loads were applied to the two-pole specimens with a 25 kN hydraulic testing machine MTS Germany, with simultaneous electrical property measurements using the abovementioned method. The cyclic compressive loading of a maximum of 2 kN corresponds to about 5 MPa in the anelastic region, and the monotonic compressive loading was completed up to damage with a loading rate of 0.1 mm/min. The recording of load and resistance values was conducted every 5 s.

## 4. Results Presentation

### 4.1. Mechanical Properties of Nano-, Micro-, and Hybrid Mortar Composites

**Figure 3:**
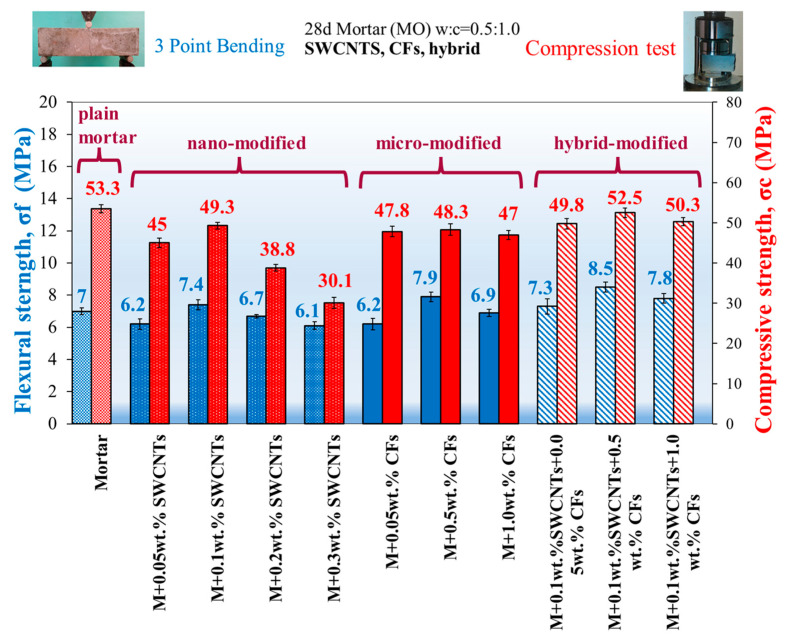
Average 28-day flexural strength, compressive strength, and standard deviations of plain mortar and strengthened mortars with SWCNTs, CFs, and hybrid reinforcement.

The 28-day flexural strength increased as a function of the nanotubes’ amount up to 0.1 wt.% of cement [10,11,13,17,18] to 0.3 wt.%, revealing the optimum content of 0.1 wt.%, and in flexural response exhibiting a 6% increase, compared to the plain mortar (Figure 3). The insufficient dispersion due to the high aspect ratio (l/h) value, as indicated in Table 1, may indicate the cause of the flexural capacity reduction even below the reference value. From the category of CFs-reinforced mortars, the optimal behavior was exposed by the 0.5 wt.% of cement, with a decrease in flexural strength of 13% compared to the reference specimen. The hybrid reinforcement (0.1 wt.% CNTs + 0.5 wt.% CFs) exhibits the expected flexural enhancement of 21%.

In contrast to flexural strength, 28-day compressive strength did not increase in all reinforced mortars, as depicted in Figure 3. Specifically, in the reinforcement with nanotubes, the compressive strength decreased from 7.5% to 43.3%. In addition, the reinforcement with CFs increased from 9.3% to 10.3%, and the hybrid reinforcement increased from an imperceptible reduction of 1.5% to 13.13%. The finest compressive strength is detected in the concentration of 0.1 wt.% SWCNTs, 0.5 wt.% CFs, and the hybrid combination of the ladder scale reinforcement. However, a reduction of up to 10% is tolerable and considered negligible. An important observation is the adequate dispersion of the 0.1 wt.% nanotubes, particularly in flexure and compressive capacity, showing the finest behavior in both mechanical strengths compared to the other concentrations. It is noteworthy that by increasing the content beyond 1.0 wt.% SWCNTs, a mechanical strength reduction is noticed due to the nanotube’s aggregation from insufficient dispersion, which leads to the concentration of agglomerates in the mortar matrix. At the points where agglomerates exist, we have a discontinuity in the cementitious material, and a concentration of stresses may be created, causing the premature failure of the specimen. Nevertheless, a positive synergetic interaction between the nano- and micro-composites is observed in the hybrid reinforcement, especially in the flexural response, as is noticed from the mechanical results [10,18].

**Figure 4:**
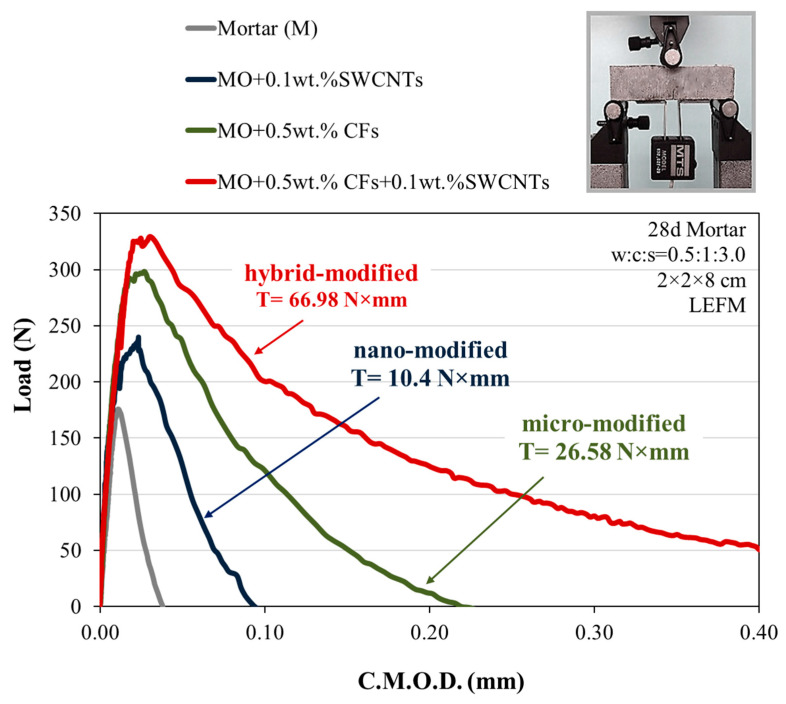
Load-CMOD curves of 28-day reference or nano-modified, micro-modified, and hybrid-modified mortars.

The flexural toughness of multi-modified mortars can be calculated due to their ability to absorb energy under the application of flexural loading, and determined from the zone under the load-deflection curve at the three midspan unnotched prisms subjected to 3-point loading, according to ASTM C1018 standards. The toughness assessment based on notched 2 cm × 2 cm × 8 cm beams is preferable to the conventional 3-point bending method, handling the specific section and a propagating macro-crack alongside the plane of the notched section [8]. The results of the experimental determination of the toughness and the energy absorption ability of hybrid multi-reinforced mortar are depicted in Figure 4. In this diagram, the load-CMOD (Crack Mouth Opening Displacement) curves are derived according to the Linear Elastic Fracture Mechanics (LEFM) test [43,44,45,52,74,75,76,77,78]. This presentation of load vs. CMOD plot results aims to highlight the optimized mechanical behavior improvement of the nano- and micro-modified and their corresponding combinations compared to the reference. At this point, it should be clarified that nano-modified mortars are those reinforced with SWCNTs, micro-modified mortars are those reinforced with CFs, and hybrid-modified mortars are reinforced with both SWCNTs and CFs. This figure shows a significant enhancement of the post-elastic response in all the multi-reinforced specimens from the load-deflection CMOD curves [57,58,59,60,61,62,63,64,65,66,67,68,69,70,71,72,73,74,75,76,77,78,79,80,81,82]. The ladder scale reinforcement improves the mortar’s load-carrying capacity and deformation ability. Specifically, the integration of 0.1 wt.% SWCNTs in the mortar matrix increased the absorbed energy, presenting an optimal toughness value of 10.4 Nmm. In the case of CFs integration, particularly at 0.5 wt.% cement, a significant elastic (pre-peak) is provided, as well as an essential improvement in the post-crack area from the first crack genesis on with T = 26.58 Nmm), demonstrating a high capability to transfer stresses and load from the matrix. Finally, the most advantageous hybrid-modified mortar combining the 0.1 wt.% SWCNTs and 0.5 wt.% CFs exhibited the finest energy absorption at a T = 66.98 Nmm, with 1509% more than the reference mortar, 921% more than the nano-modified mortars, and 544% more than the micro-modified mortars. All types of reinforcement improved the flexural toughness by about an order of magnitude compared to the reference specimens.

The independent dimensions’ ratios of the toughness of the modified mortars divided by the toughness of the reference specimen (T_mod_/T_ref_), as well as the ratios of the maximum applied load (Load_FC_/Load_PL_) and CMOD deflection at first per those of the peak load (CMOD_FC_/CMOD_PL_), respectively, of the reference specimen and nano-, micro-, and hybrid-modified 28-day mortars, are listed in Table 2. The ratio (T_mod_/T_ref_) indicates the increase in toughness compared to the reference mortar. It is obvious that when the reinforcement passes from nano- to micro-scale, the ratio increases by 149%, with the optimal hybrid-modified mortar exhibiting an augmentation of 528% compared to the nano-modified specimen. It is worth noting that a gradual decrease of the Load_FC_/Load_PL_ and CMOD_FC_/CMOD_PL_ ratios for the three types of reinforcement, from nano to hybrid reinforcement, is observed compared to the largest ratio of the reference specimen. The hybrid-modified mortar demonstrated the optimal indices with a drop of 25% for the index Load_FC_/Load_PL_ and 79% for the CMOD_FC_/CMOD_PL_ ratio, compared with the unreinforced mortar. This fact demonstrates that the reinforcement efficiently transfers load from the cementitious matrix, contributing to the elastic pre-peak of the augmentation load, detaining and eliminating crack development at multiple levels, and exhibiting post-cracking performance after failure. The synergistic effect of the multiscale reinforcement contributes to the improvement of the elastic region, but even more so the post-elastic region, as the microfibers exhibit enhanced deformability.

### 4.2. Multi-Modified Mortar Self-Sensor Performance

#### 4.2.1. Electrical Conductivity

**Figure 5:**
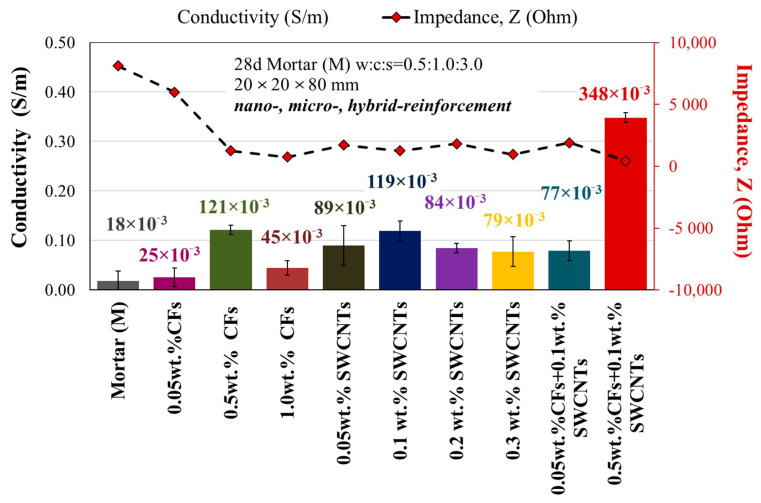
Average conductivity of 28 d plain reference and modified mortars with different concentrations (wt.%) of SWCNTs and CFs, or hybrid combination at 28 days.

Three specimens for each type of modified mortar were used to measure their electrical resistivity, which is then converted to conductivity. The average electrical conductivity and impedance of three 28-day multi-modified mortars with different concentrations of carbon nanotubes or microfibers and the hybrid combination are presented in Figure 5. The 0.5 wt.% CF modified mortars had an increased electrical conductivity of 121 × 10^−3^ S/m, which was an order of magnitude greater (572% augmentation) than the reference specimen, but also from the mortars strengthened with 0.05 wt.% and 1.0 wt.% microfibers. The nano-modified mortars presented an improved electrical conductivity in all cases, especially in the content of 0.1 wt.% SWCNTs (119 × 10^−3^ S/m) increased by 561% compared to the reference specimen. Finally, in the hybrid-modified case, the optimal nano- and micro-reinforcement combination demonstrated exceptional electrical conductivity, with 348 × 10^−3^ S/m and an 1833% improvement over the plain mortar. As with mechanical and electrical properties, the modified mortars containing 0.1% SWCNTs and 0.5% CFs and their combination demonstrated the most advantageous behaviors. The reason is that in the larger contents, a concentration of fibers and agglomerates was observed, creating pores in the mass of the matrix. This fact results in a mechanical performance decline, and the electrical properties are reduced; however, when the optimum reinforcement is reached, both mechanical and electrical behavior are enhanced [10,18,60,61]. 

#### 4.2.2. Self-Sensing Ability of Multi-Reinforced Mortars

**Figure 6:**
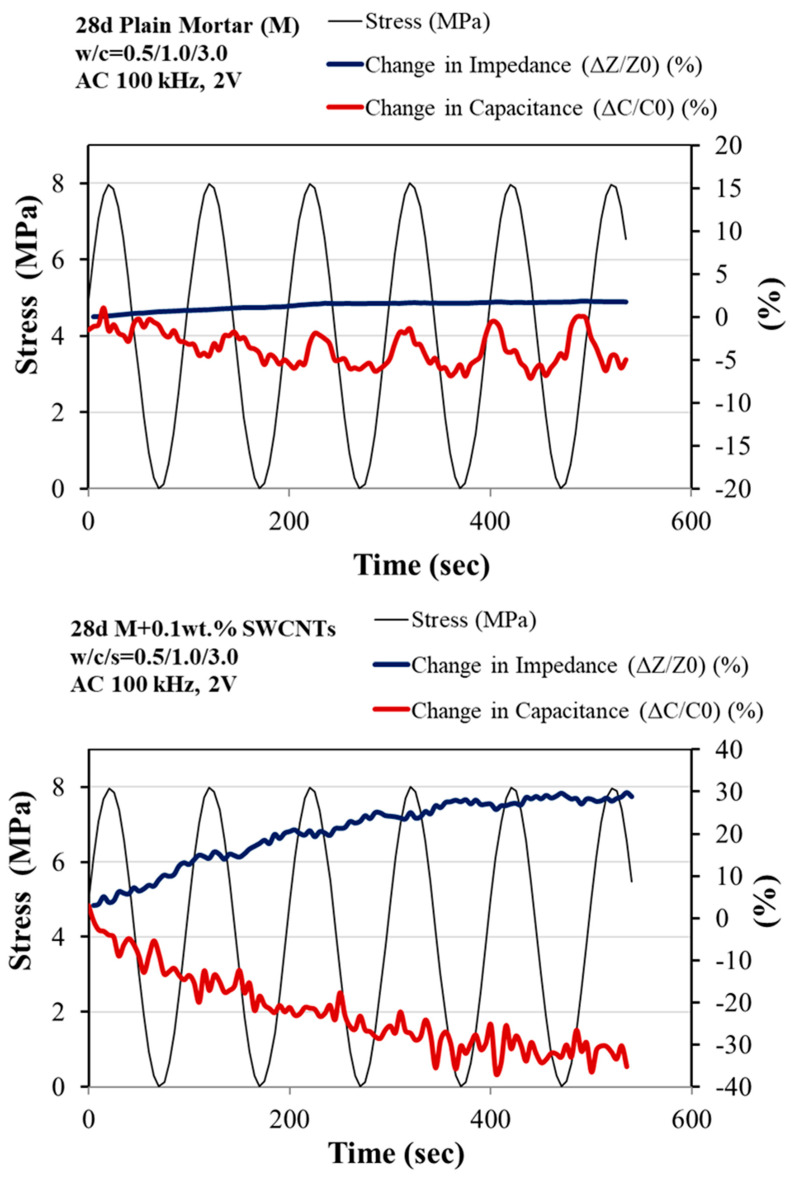

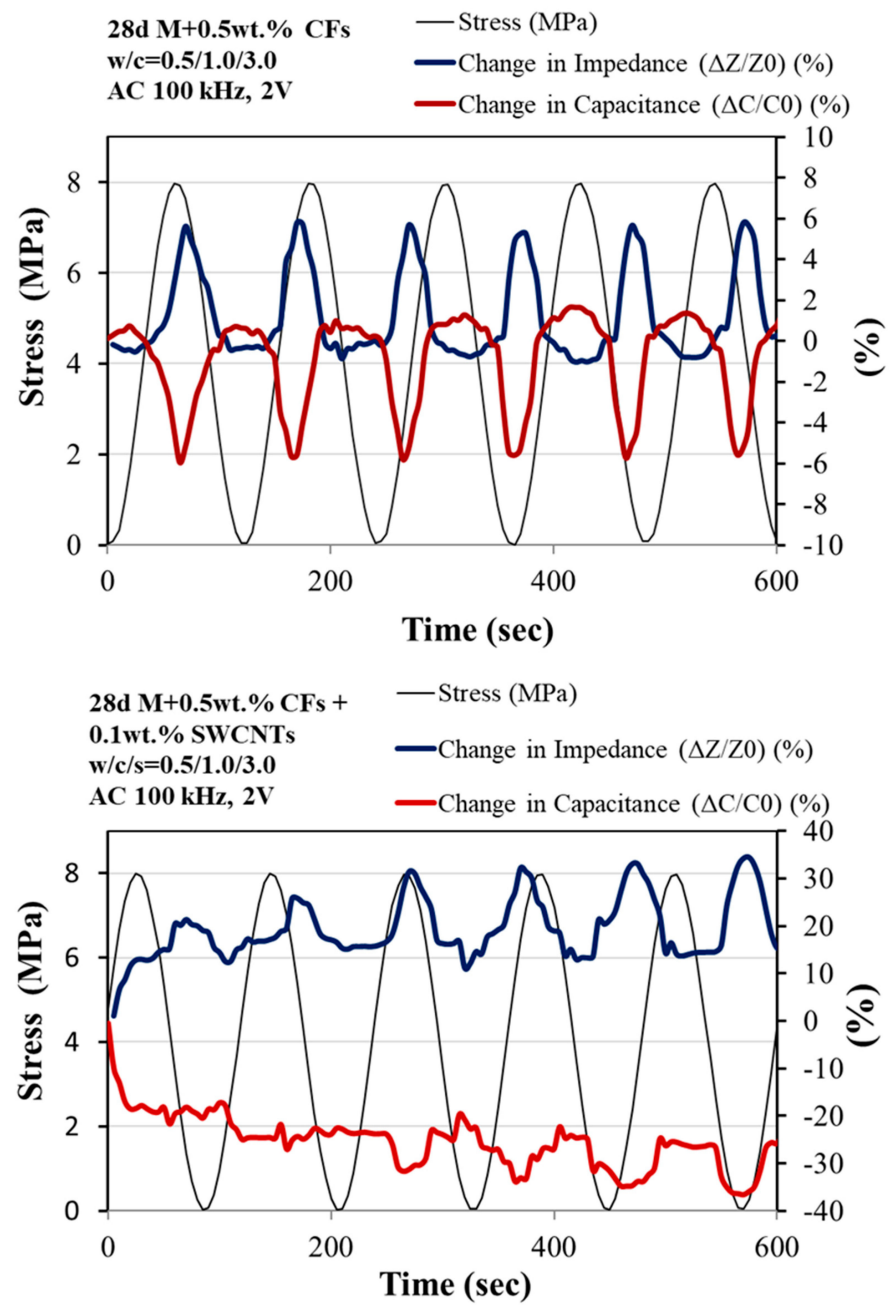
Piezoresistive responses under cyclic loading of 28--d plain, reinforced with 0.1 wt.% SWCNTs, with 0.5 wt.% CFs, and hybrid-modified mortars.

**Figure 7:**
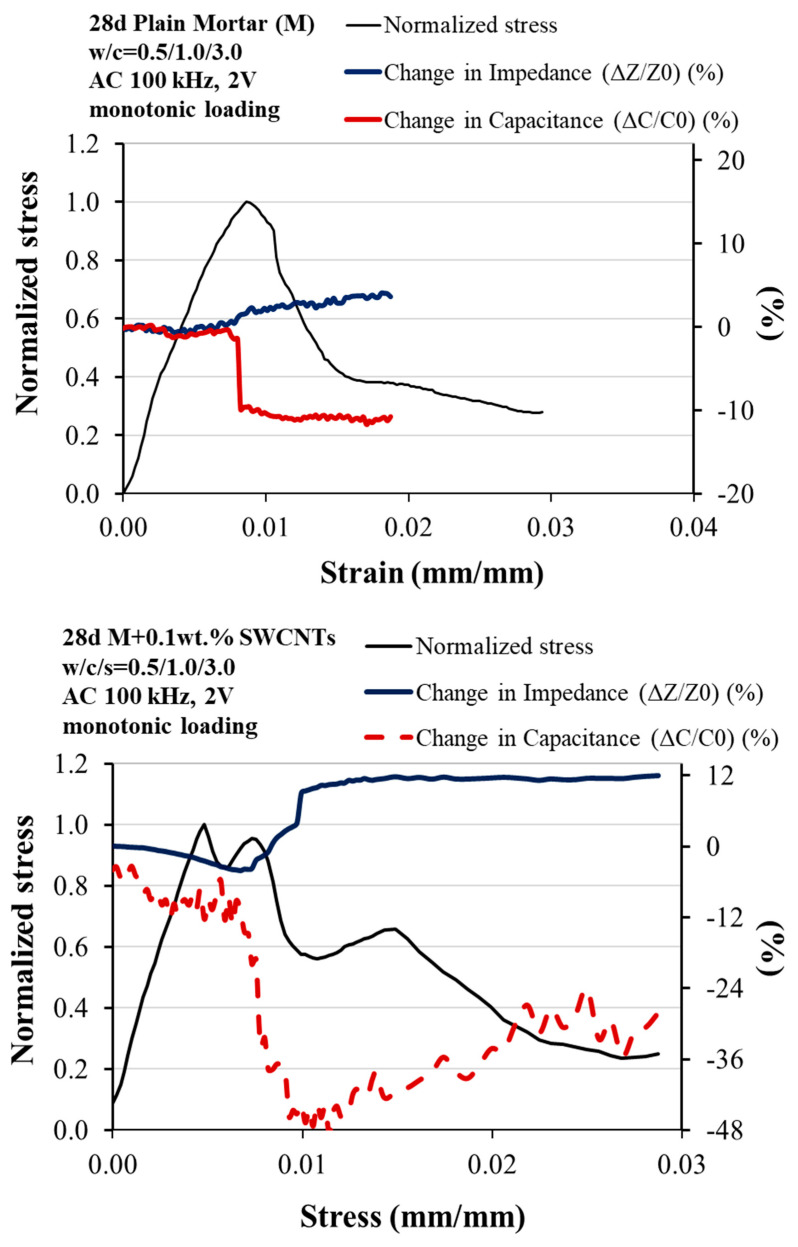

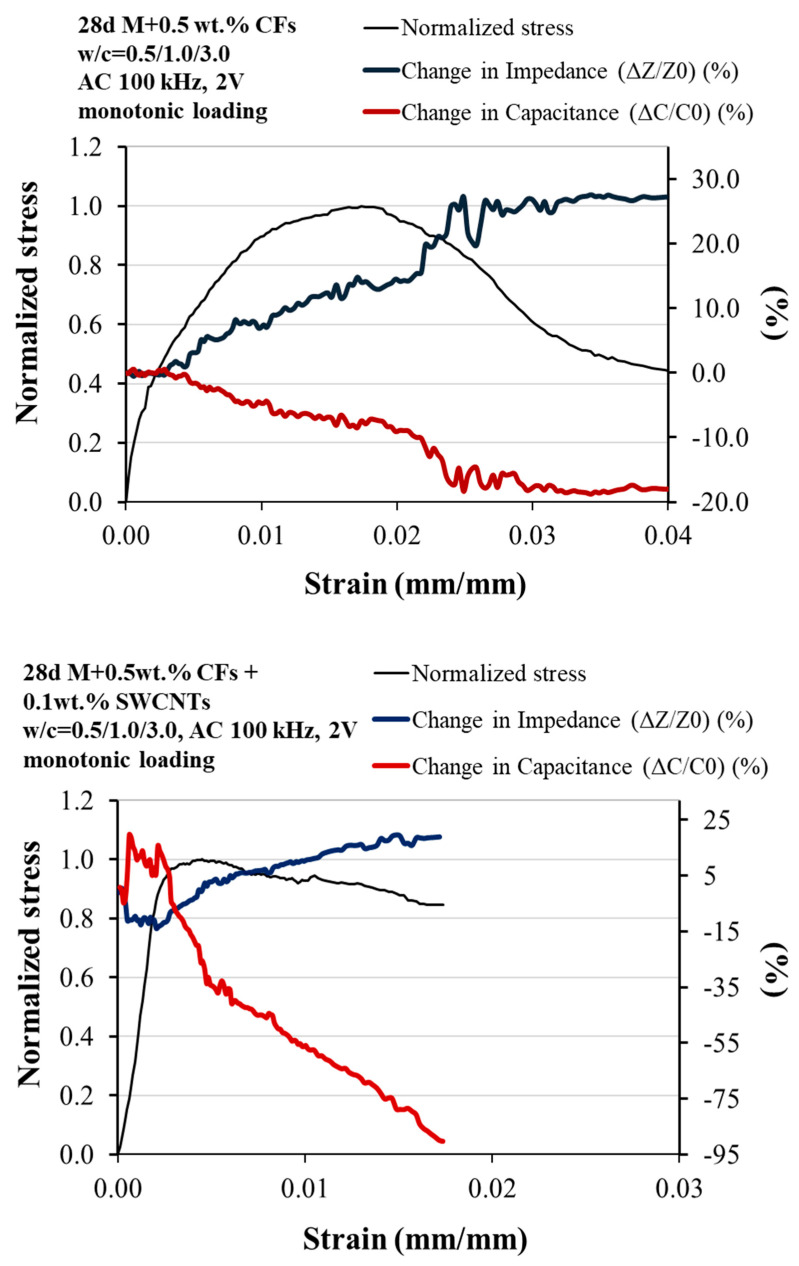
Piezoresistive response under monotonic compressive loading of 28-d plain, reinforced with 0.1 wt.% SWCNTs, with 0.5 wt.% CFs and hybrid-modified mortars.

The piezoresistivity values are used to assess the self-sensing ability of ladder-scale reinforced mortars. It is studied according to the fractional changes in electrical resistance under synchronized uniaxial compressive loading. The cyclic loading in the elastic region and the monotonically increasing load up to failure with simultaneous electrical measurement are illustrated in Figure 6 and Figure 7. Figure 6 represents the compressive stress over time, that is, the piezoresistive behavior of 2 cm × 2 cm × 8 cm multi-reinforced mortar with SWCNTs and CFs at different volume fractions of cement under cyclic compressive loading within the pre-peak region (0–8 MPa). All the multi-modified mortars exhibit an enhanced piezoresistive response as their change in impedance gradually increases, and their change in capacitance progressively decreases while loading, although both increase and decrease, respectively, with unloading. Because of the similarity with the electrical response, the multi-modified mortars exhibited improved strain sensing responses when the optimum nano-reinforcement (0.1 wt.% SWCNTs), the micro-reinforcement (0.5 wt.% CFs), and the hybrid combination of them were added and incorporated (Figure 5).

Figure 6 depicts the changes in impedance and capacitance for optimum multi-reinforced mortars with simultaneous monotonic compressive load application up to specimen failure. It is noteworthy that the phenomena observed in the electrical measurements so far (conductivity, piezo-resistivity) are still evident in the monotonic application of compressive load. The larger the mortar amplification scale from nano to micro, the greater the changes in the electrical measurements of impedance and capacitance. That indicates the self-sensing ability of all the modified specimens, particularly the hybrid reinforced mortars.

Table 3 shows the average change of impedance, capacitance, and resistivity over 28-day plain and hybrid-modified mortars. CFs micro-reinforced mortars improved by 137%, 119%, and 103%, respectively, compared to the 0.1% nano-level sensor. Furthermore, hybrid-level strengthening demonstrated improved piezoresistive behavior and linear variation of sensitivity, as well as effectiveness in tree ratios of up to 289%, 324%, and 576%, respectively, when compared to nano-modified mortars, and 64%, 93%, and 234%, respectively, for micro-modified mortars. This finding follows previous investigations of reinforced mortars with carbon fibers, as examined by our previous work and that of other researchers [10,18].

### 4.3. AC-IS Response of Nano-, Micro-, and Hybrid-Modified Mortar

AC impedance spectroscopy (ACIS), considered a potential non-destructive method, is a category of the fundamental theory of impedance spectroscopy (IS) and is used for interfaces between solids or solids and liquid systems [79,80,81,82]. The frequency area varies between Hz and MHz, while in this study, the measurement range is between 0.1 and 200 Hz. The equation between voltage (V), current, and resistance (R) for direct current (DC) could be defined using Ohm’s law as follows (Εquation (1)):

V = I × R (1)

Nevertheless, for alternating current (AC), there is a time dependency for the input voltage (V) and output current (I), hence altering Ohm’s law to the Εquation (2), where the impedance Z(ω) derives from Εquation (3).

V(t) = I(t) × Z(ω),(2)

Z(ω) = V(t)/I(t) = E0 × sin(ωt) × I0 × sin(ωt + θ),(3)

where modulus |Z(ω)| = |Z|/|I|× ω and θ(ω) is the phase angle [80,81,82].

**Figure 8:**
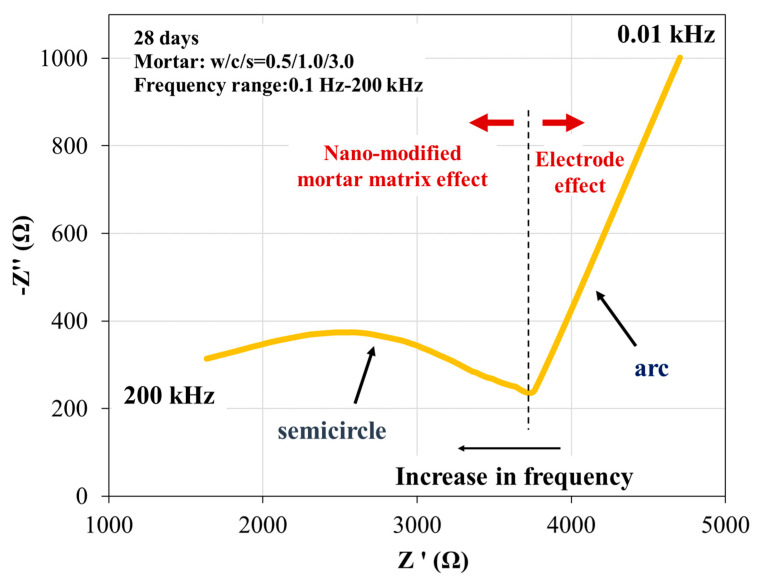
A typical Nyquist plot of a 28-day nano-reinforced cement mortar matrix with 0.3% carbon nanotubes (SWCNTs).

Impedance consists of the assumption of the real part of impedance (resistivity (Z’)) and the imaginary part of impedance (reactance (Z″)). Nyquist plots are generally utilized for cementitious materials to demonstrate, using a visual image, the equations of the ACIS, expressing the relations between the real and imaginary parts of the ACIS in a specific frequency range. This two-point measurement concerns the application of AC excitation of various frequencies to the mortar matrix, providing reliable results [65,66,67,68,69]. The Nyquist plot compares the negative imaginary part of impedance, Im(Z), to the real part of impedance, Re(Z), of the matrix’s measured response, with frequency increasing from right to left. Semicircles or arcs are designed in the plot whose diameters reflect the resistances of the different mortar specimens. A typical Nyquist plot for 28-day nano-modified mortar reinforcement is depicted in Figure 8. In this diagram, an arch on the left considers the simultaneous electrical response of the titanium electrode. The semicircle on the right (bulk arc), called a capacitive loop, combines the resistance and capacitance of the specimen. Every specific value on the capacitive loop signifies the impedance value at the corresponding frequency.

**Figure 9:**
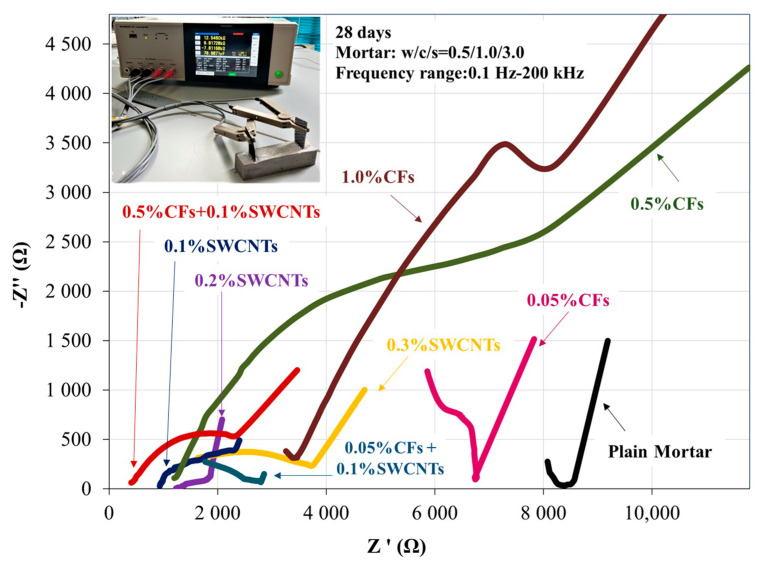
Nyquist plot of a 28-day nano-, micro-, and hybrid-reinforced cement mortar matrix with different concentrations of carbon nanotubes (SWCNTs) and carbon microfibers (CFs).

In Figure 9, a Nyquist plot of the imaginary part vs. the real part of the impedance of a 28-day nano-, micro-, and hybrid-reinforced cement mortar matrix with different concentrations of carbon nanotubes (SWCNTs) and carbon microfibers is depicted. At first glance, there appears to be a one-order-of-magnitude increase in the imaginary part (-ImZ) of the impedance for all nano- and micro-modified specimens compared to the reference mortar. Specifically, the 0.1 wt.% nano-modified mortar appears to have a low imaginary part (Ζ″) and a low real part (Ζ′) compared to other nano-reinforced specimens and obviously to the plain cement mortar. In the micro-reinforcement, the incorporation of the 0.5% CFs provides a significantly high imaginary part (Ζ″) and low real part (Ζ′) compared to plain and nano-reinforced specimens. Finally, the hybrid combination of these two best ladder scale reinforcements resulted in the best response of all modified mortars. Thus, it presents a lower resistivity (Z′), indicating higher conductivity than the modified mortars. Furthermore, when compared to the rest of the modified mortars, the low magnitudes of the reactance (Z″), which correspond to the imaginary values, indicate capacitance ability [60,61,82].

This experimental investigation contributes significantly to creating cementitious mortar sensors with enhanced mechanical performance and advanced electromechanical properties simultaneously. Results detect that the beneficial ladder scale hybrid-modified specimens using nanotubes and micro-scale carbon fibers exhibit good electrical conductivity and piezoresistive performance at the multiscale and improved energy absorption, preventing the genesis and propagation of micro- and macro-cracks.

## 5. Conclusions

The mechanical (flexural, compressive, and toughness) and electrical effects (conductivity, real and imaginary parts of impedance, resistivity, and capacitance), as well as their combination expressed by the piezoresistive response of nano, micro, and hybrid reinforcement, are experimentally investigated in this research. The consequences demonstrate that the optimum mortar reinforcement at the nanoscale is the concentration of 0.1% SWCNTs, and similarly, at the microscale the 0.5 wt.% CFs exhibited a marginal reduction in compressive strength but an extraordinary improvement in flexural strength, flexural toughness, and electrical response as an optimum concentration between 0.05 wt.% and 1.0 wt.% CFs. This fact motivates further investigation into the hybrid combination of the best multi-reinforcement, while demonstrating their synergistic action in electromechanical properties. The major issues are listed as follows:

(1) The concentration of 0.1 wt.% SWCNTs in the nanoscale, the 0.5 wt.%, CFs, and the hybrid reinforcement exhibit the finest flexural strength compared to plain cementitious mortar. Marginal reduction in the compressive strength of the modified mortars within acceptable limits compared to the reference specimens;
(2) The elastic (pre-peak) and post-elastic responses are significantly enhanced in all the reinforced mortars by about an order of magnitude compared to the reference specimens. The hybrid-modified mortar exhibited the finest energy absorption, with a value of 1509% more than the reference. The hybrid-modified mortar demonstrated the optimal indices with a drop of 25% for the index Load_FC_/Load_PL_ and 79% for the CMOD_FC_/CMOD_PL_ ratio, compared to the unreinforced mortar;
(3) The electrical conductivity and impedance of multi-modified mortars with all the different concentrations of carbon nanotubes, microfibers, and the hybrid combination increased by an order of magnitude compared to the reference mortar;
(4) The change rate of impedance and capacitance in the hybrid-level strengthening demonstrated improved piezoresistive performance with three ratios of up to 4289%, 324%, and 576%, respectively, compared to nano-modified mortars, and 64%, 93%, and 234%, respectively, compared to micro-modified mortars.

In conclusion, the results verify the dual role of multi-reinforcement. Consequently, over the electrical measurements and piezoresistive performance, it could be detected that it consists of an index to assess the crack’s genesis, health monitoring, and stress/strain sensitivity, recognizing the change in the applied load or induced mechanical deformation. Hence, they can be considered to be utilized as piezoresistive sensors. This research anticipates underwriting significantly to create cement mortars with promising electromechanical properties.

## Figures and Tables

**Table 1:** Properties of single-walled carbon nanotubes (SWCNTs) and microfibers (CFs).

Type	Carbon Content (%)	Tensile Strength (MPa)	Tensile Modulus (GPa)	Strain at Failure (%)	Density (gr/cm^3^)	Filament Diameter (μm)	Length (mm)	Aspect Ratio
SWCNTs	99	50–100	1000	-	1.87	1.6 ± 0.4	>5	2500
CFs	95	4.9	230	2.1	0.18	7	6	858

**Table 2:** First crack load and toughness values of reference mortar and nano-, micro-, and hybrid-reinforced cement mortars with SWCNTs and/or CFs.

Sample	Τ_mod_/T_ref_	Load_FC_/ Load_PL_	CMOD_FC_/ CMOD_PL_
Reference Mortar	-	0.93	0.81
M + 0.1 wt.% SWCNTs	2.56	0.84	0.42
M + 0.5 wt.% CFs	6.39	0.76	0.32
M + 0.5 wt.% Cfs + 0.1 wt.% SWCNTs	16.1	0.70	0.17

T_mod_ = Toughness of modified mortar, T_ref_ = Toughness of reference mortar, FC = First crack, PL = Peak load.

**Table 3:** Changes in impedance, capacitance, and resistivity values (%) of 28 d plain and hybrid-modified mortars.

Reinforced Mortars	Change in Impedance ΔZ/Z_0_ (%)	Change in Capacitance ΔC/C_0_ (%)	Change in Resistivity Δρ/ρ_0_ (%)
0.1 wt.% SWCNTs	2.41	2.73	2.56
0.5 wt.% CFs	5.72	5.99	5.19
0.1 wt.%SWCNTs + 0.5 wt.% CFs	9.38	11.58	17.32

## Data Availability

The data presented in this study are available on request from the corresponding author.
